# Sports training in virtual reality with a focus on visual perception: a systematic review

**DOI:** 10.3389/fspor.2025.1530948

**Published:** 2025-03-20

**Authors:** Kerstin Witte, Dan Bürger, Stefan Pastel

**Affiliations:** Department Sports Engineering/Movement Science, Otto-von-Guericke-University Magdeburg, Magdeburg, Germany

**Keywords:** virtual reality, sports training, visual perception, motor learning, literature review

## Abstract

**Introduction:**

There is an increasing endeavor to use Virtual Reality (VR) technologies in sports training. Because visual perception is crucial for sports performance, it should also be considered in the development of VR tools. The aim of this review is to summarise the literature on the application fields and domains of sport in VR and to examine what needs to be considered when developing such VR training tools with regard to visual perception and its manipulation. Furthermore, it is still unclear to what extent the user's body or that of their teammates or opponents must be visualized in VR.

**Methods:**

The literature search was conducted based on five databases: PsychInfo, Pubmed, Scopus, SportDiscus, and IEEE Explore. Review articles and original articles written in English and published between 1 January 2015 and 31 March 2024 were considered.

**Results:**

Through a qualitative analysis of the selected research, we identified 12 reviews and 46 research articles. While the applications of VR in sports initially focussed on endurance sports at the beginning of the development of VR tools, within the last 5 years VR was found in almost all sports, such as team sports, martial arts, and individual sports. The most common sports domains in which VR is used are motor learning, training of visual perception, decision making processes, and anticipation skills, as well as many sport-specific trainings. The review provides an overview of the extent to which visual aids in VR can be used to optimize motor learning and training, also taking into account the transfer to the real world.

**Discussion:**

VR offers many opportunities to visually support sports training and motor learning. It has been shown that training in VR based purely on visual perception can be successful in many sports and sports domains. The following visualization options are already being used: different viewing perspectives, variation of the demonstrated speed of movement, use of virtual mirrors, visualization of the necessary body parts, visual manipulation to identify important stimuli, display of movement trajectories, and graphic aids. In future, more studies should be conducted to compare training under virtual conditions with training under real conditions and to investigate transfer effects. Eye tracking should also be used for this purpose in order to compare visual perception in VR and in reality. Less is known about how VR training can be realized in terms of frequency, duration, and intensity and how VR training can be combined with training in the reality world.

## 1 Introduction

Athletes, coaches, and other sport-related professionals are increasing the use of virtual reality in sports (1). The authors define VR applications in sports as the use of computer-generated sport-relevant content and a means for the athlete to interact with the virtual environment. Duan (2) presents an application of VR technology based on computer vision in exercise systems and verifies that the system can improve the effectiveness of sports exercise. VR simulators are gaining traction for practical purposes as well, such as managing overcrowded match schedules and adhering to training load restrictions aimed at minimizing injuries (3). While the use of VR tools allows better control of visual stimuli, immersion, and interaction with the environment compared to video simulations, the underlying mechanisms of how experts use visual information for anticipation are not sufficiently known and included in the current design of VR tools.

In order to ensure positive transfer effects of sports training in VR to the real environment (RE), it is necessary to understand that motor behavior in VR may well differ from that in a RE. For example, research by Hollmann et al. (4) suggest based on parameters determined from the time courses of the vertical ground reaction forces, that walking in a virtual environment can cause gait instability in healthy subjects. One possible reason for this is that visual perception (VP) was impaired with the technology used [Elumens VisionStation® 1024 (Elumens Corporation, Durham, NC) projection system], in contrast to today's head mounted displays (HMDs). However, even with modern VR devices, the limitations compared to real vision described in the following section must be kept in mind. An important factor for realistic HMDs is their field of view (5): The human binocular field of view is approximately 200° horizontally by 100° vertically, with a binocular overlap of 120°. An overview of the field of view parameters of different HMDs was created by Sauer et al. (6). In contrast to the official specifications of the manufacturers, Sauer et al. (6) determined smaller fields of view depending on the type of HMD (e.g.,: HTV Vive Pro 94° horizontal and 91° vertical, official specification: 110°) (6). However, these limits are being pushed further by increasingly modern technologies. Another difference between natural vision and vision using an HMD is the resolution (human eye: ca: 32,000 × 24,000 pixels, HMD: on average: 1,440 × 1,600 pixels) (7). Further limitations of HMDs are described in (8). Based on the image calculation using two virtual cameras, the position of these cameras is decisive based on the eye distance set in the HMD (interpupillary distance). Even small differences in the distance between the virtual cameras can lead to major effects in distance estimation. In the past, this often led to the distance in VR being underestimated (9). However, not all studies can prove a reduced distance estimation in VR, especially newer ones that refer to advanced HMDs (10).

The authors' practical experience has shown that, despite using state-of-the-art technologies, it is still impossible to achieve the same field of vision and sufficient pixel density as in natural vision. Furthermore, the vergence-accommodation effect is part of a neurophysiological control circuit that is affected by VR technology. This convergence-accommodation conflict can also lead to incorrect depth perception (11), eye fatigue, and dizziness. The depth of field perceived in the real environment is caused, among other things, by the fact that objects at a greater distance appear blurred. Possible technological solutions could include the use of optical liquid crystal elements with a thin form factor for light modulation (12).

Despite this current visual limitation of HMDs, the manipulation of VP, standardization, and human interaction with the virtual environment offer many advantages that can be used in sports training and sports research. These advantages can be used to enable the transfer of VR training to the real world. Nevertheless, there is still no clear evidence in the literature that movements learned and trained in VR are directly transferable to RE (1, 13). One possible reason could be the different mechanisms of VP in both environments (13). However, in order to develop efficient VR training tools, it is important to know what to consider for VP in VR for the various specific application areas and sports.

A lot of applications of VR technologies in sports relate to game sports and combat sports. It was found that special VR tools can help to train anticipation and reaction skills und lead to better performance in RE [e.g., baseball (14) and karate (15)]. Such training tools require special demands on the developer. On the one hand, a virtual environment that is as natural as possible requires a realistic visualization of sport-specific equipment and locations. On the other hand, realistic visualizations of other athletes, teammates, opponents, and coaches are also necessary for sporting interactions, for example in team sports and martial arts. The creation of interactive characters poses a particular technological challenge (16).

It can be established that VR tools have great potential to support the learning and training of skills in many sports. However, it is important to be aware that multisensory conflicts can occur, which result in particular from the fact that the environment is generally only virtualized visually (17). Despite these limitations and the challenges in achieving natural visualization, there have already been successful applications in sports training (15, 18, 19), although they remain relatively few in number.

## 2 Objectives

Several studies (1, 5, 15, 19) have already shown that VR training tools can support athletic training and have a positive influence on athletic performance, despite the limitations mentioned above.

The present review is intended to address the requirements of a VR training tool in a sporting context. An essential point of reference is the work of Pastel (20), which deals with motor behavior and basic movements in virtual environments and examines defined eye movement tasks, visualization of one's own body in various motor tasks, spatial orientation, and distance estimation. This review will focus on the specific applications of VR in each type of sport to explore and understand how visualization is used in these contexts. The visualization methods found should help to find the essentials of VR training tools that contribute to optimizing new VR training tools.

This review is intended to address three objectives.

Objective 1: Identify in which sports domains and sports disciplines VR has been used with consideration of the visual components.
The following sports domains will be focussed on in particular: training, tactics, decision-making processes, reaction capability, anticipation skills, and learning sport-specific techniques.
Objective 2: Identify requirements for the visualization of the user's body (21, 22), and that of the other players in VR.
Objective 3: Determine which visualization options were used to successfully transfer the effects of VR training to athletic performance in RE and whether VR training alone is sufficient.

## 3 Methodology

**Figure 1:**
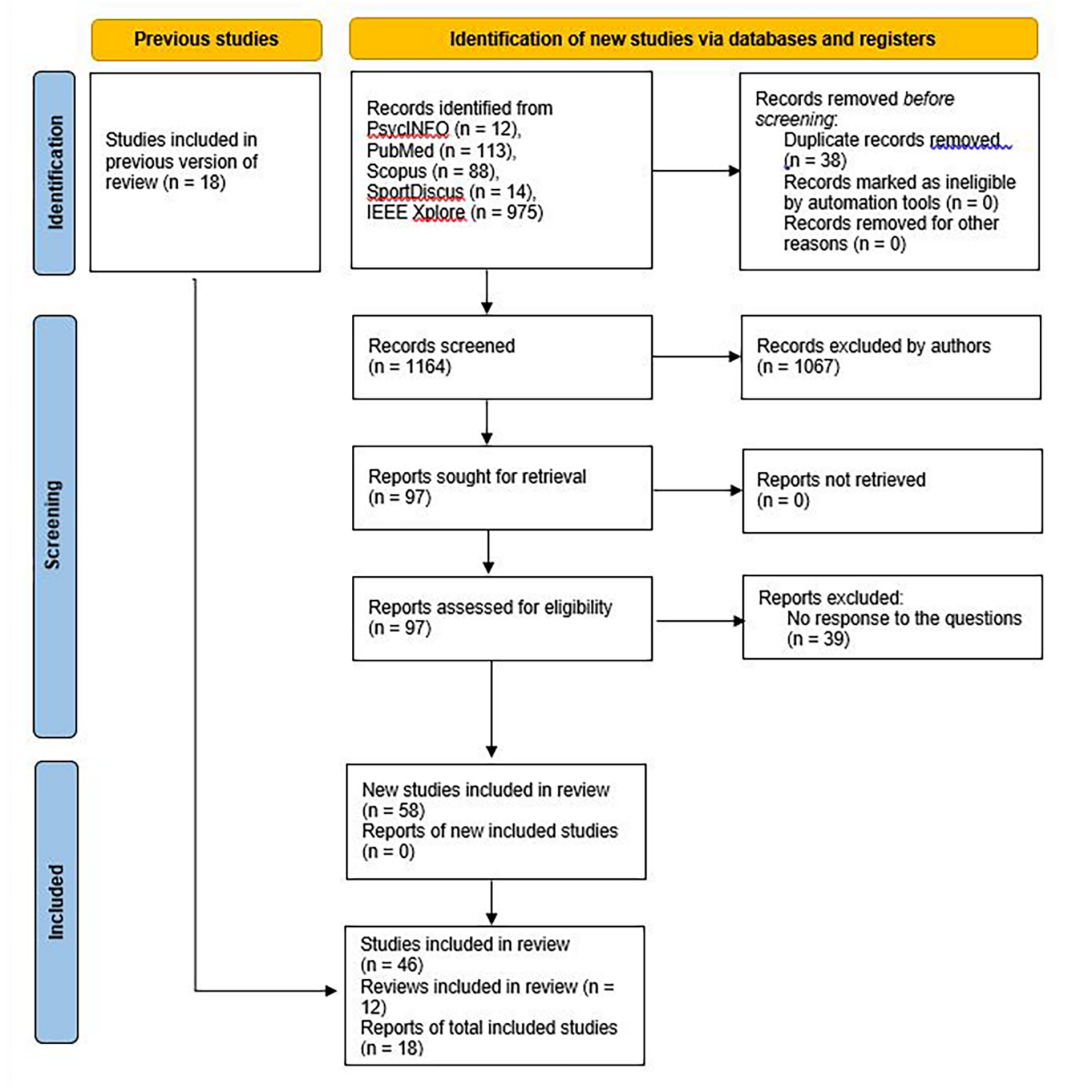
PRISMA flowchart.

The systematic review procedure is based on the PRISMA flowchart (Figure 1). The literature search was conducted based on five databases: PsychInfo, Pubmed, Scopus, SportDiscus, and IEEE Explore. The following keywords were used: “virtual reality” OR “virtual environment” AND “visual perception” AND “sport” AND “healthy” “human” OR “healthy subjects” OR “healthy adults”. The publication period was set from 1 January 2015 to 31 March 2024. This temporal limitation was intended to ensure that only modern VR technologies (primarily HMD) were considered so that visual perception VP deficits due to outdated technologies were not included. Further inclusion and exclusion criteria are contained in Table 1.

**Table 1:** List of inclusion and exclusion criteria.

Criteria	Inclusion	Exclusion
Language	English	All other languages
Population	Athletes, healthy young people or adults	children (<10 years) older adults (>70 years) patients with orthopaedic or cognitive problems
Study type	Original Research and Reviews studies in peer-reviewed journals,	Narrative synthesis
Scope	Virtual reality, Sports	Augmented reality, Rehabilitation, Clinical analysis

A total of 1,164 articles were found. After the deduction of 1,067 duplicates, a further evaluation of the year of publication, title, English language, and the availability of the complete article, 97 articles were included in the analysis. No automated tools were used. The search was conducted by the first author in its entirety. The co-authors performed random samples, searched for duplicates, and checked the articles found with regard to the inclusion and exclusion criteria (Table 1). Analyses of the full texts concerning the research questions resulted in a final number of 58 articles. These included 12 overview articles or literature reviews and 46 research articles.

## 4 Results

The articles found in this review are analyzed below. A distinction was made between overview articles respectively literature reviews and research articles.

### 4.1 Analysis of overview articles and literature reviews

Table 2 contains the twelve review articles and overviews found, which are explained in more detail below.

**Table 2:** Overview articles and literature reviews dealing with visual perception in VR and sport (authors in alphabetic order).

Author(s)	Year	Title	Topics/Questions	Key findings
Appelbaum and Ericson (23)	2016	Sports vision training: A review of the state-of-the art in digital training techniques	• Use of digital sports vision training tools is new • Identify and categorize existing approaches	• Virtual reality simulations can recreate and enhance sports contexts to promote certain sport-specific visual and cognitive skills.
Gerschütz et al. (24)	2019	A review of requirements and approaches for realistic visual perception in virtual reality	• Current use of VR technology in the product development process and outline of recent VR hardware developments	• Discussion of various approaches to optimize the visual perception in detail
Harris et al. (25)	2019	Virtually the same? How impaired sensory information in virtual reality may disrupt vision for action	• Aims to answer the question of how the novel perceptual environment of VR can influence vision for action by leading users away from a dorsal control mode	• A displacement from dorsal to ventral action control can result in a fundamental discrepancy between virtual and real capabilities, which has important consequences for our understanding of perception and action in the virtual world.
Hoang et al. (26)	2023	A Systematic Review of Immersive Technologies for Physical Training in Fitness and Sports	• Research how immersive technologies are used for physical exercise in fitness and sports	• Recommendations for future work and highlight the need to support better collaboration with industry partners, trainer-trainee experiences, and social dynamics of sports for designing better experiences. • Improving motivation to exercise was a big theme that we identified through the review
Mahalil et al. (27)	2020	A literature review on the usage of Technology Acceptance Model for analysing a virtual reality’s cycling sport applications with enhanced realism fidelity	• Determining the use of the Technology Acceptance Model (TAM) to validate the acceptance of VR-based exercises with a special focus on sports cycling	• It was found that all researchers use the basic elements of realism fidelity: visual, audio, and interaction. These three elements are found to be efficient among athletes. • When the athletes experience high realism fidelity in VR, the athlete’s acceptance of using the system for training is anticipated to increase.
Malachi et al. (28)	2024	A Systematic Literature Review of Virtual Reality Implementation in Sports	• What are the purposes of using Virtual Reality in sports? • Which type of virtual reality devices are used in sport? • What is the approach of virtual reality as user experience in sport?	• Most purpose used: physical training. • Three types of VR tools: StandAlone VR, Mobile VR, and PC VR.
Müller et al. (3)	2023	Attributes of Expert Anticipation Should Inform the Design of Virtual Reality Simulators to Accelerate Learning and Transfer of Skill	• The purpose of this brief opinion paper is to discuss what is currently known about expert pick-up of visual information for anticipation and how it can be trained, with a view that this knowledge can better inform VR simulator content design.	• The most important mechanisms underlying the visual anticipation of sports experts are explained and how this knowledge can be incorporated into the development of virtual reality simulators to better assess and train anticipation skills. • For the development of VR simulators, it is necessary to include the mechanisms of visual anticipation of experts; as otherwise, these systems could impair or compromise the performance of athletes.
Neumann et al. (1)	2018	A systematic review of the application of interactive virtual reality to sport	• Documentation of research on the application of VR to sport	• The majority of research has been conducted on endurance sports, such as running, cycling, and rowing, and more research is required to examine the use of interactive VR in skill-based sports.
Pastel, Marlok et al.	2022	Application of eye-tracking systems integrated into immersive virtual reality and possible transfer to the sports sector—A systematic review	• Review about the possibilities of integration of eye tracking in VR to improve usability, interaction methods, image presentation, and visual perception analysis in future physical training scenarios.	• The existing methods are feasible due to the performance of the integrated eye-tracking systems but still need to be improved for practical use in sports.
Putranto et al. (29)	2023	Implementation of virtual reality technology for sports education and training: systematic literature review	• Summary of findings on the introduction of VR, particularly in physical education, as a training tool to improve the skills of athletes and overcome the shortcomings of traditional teaching methods.	• Virtual Reality is used to improve the individual performance of athletes • Problems with HMDs can be solved • VR in combination with Motion Capture
Richlan et al. (30)	2023	Virtual training, real effects: a narrative review on sports performance enhancement through interventions in virtual reality	• Qualitative overview and narrative summary of such studies to clarify the potential benefits of VR technology for sports performance enhancement, to extract the main characteristics of the existing studies, and to inform and guide future research.	• Interventions in VR have the potential to achieve real effects in improving sports performance through the training of motor and psychological skills and abilities of athletes, including perceptual and behavioral skills, strategic, tactical, and decision-making decision making, reaction to unexpected events, and improving mental resilience and mental performance under pressure.
Yuqing et al. (31)	2021	VR Technology and Application in Martial Arts	• The purpose of this study is to collect and summarize the application of martial arts in VR from multiple perspectives, aiming to classify the virtual martial arts system according to the feedback mode provided by the Virtual Environment (VE), the function mode of martial arts and the key needs of users. • The review is organized according to three perspectives: feedback mode, function mode, and user mode.	• Review work is useful for both researchers and educators to develop virtual martial arts systems for enhancing the protection and inheritance of martial arts. • Discussion of different visual feedback forms

The literature review by Mahalil et al. (27) found that the acceptance of VR technology in sports is often validated through the use of the Technology Acceptance Model (TAM). Based on their analysis, it can be assumed that if the athletes experience a high degree of realism in VR, their acceptance of using the system for training is likely to increase. After these general comments on VR training tools, the results of the reviews on the three objectives will be reported.

#### 4.1.1 Objective 1: sports domains and sports disciplines

The review by Neumann et al. (1) concluded that in the earlier period between 2000 and 2016, the most common applications in sports were cycling, running, rowing, weightlifting, and golf. It should be noted that these sports contain elements of endurance and persistence. Such sports can be relatively uncomplicated and translate into a virtual environment (1). The authors propose a model of interactive VR in sports and sport-related exercises showing the relationship between components of the VR system, current outcomes, and post-task outcomes. They recommend for future studies to apply VR technologies also in skill-based sports. In particular, more research is needed into the effectiveness of VR for learning and training to improve sport-specific skills. As the relatively recent review by Hoang et al. (29) documents, game sports are also increasingly using VR tools. The authors (29) found out that the usage of VR in football, basketball, or table tennis users could improve their health and sport-specific skills.

Hoang et al. (26) highlighted the increasing number of publications on the use of VR in physical training in fitness regarding different goals: Improvement of performance (46 papers), motivation (33 papers), engagement (26 papers), and training (26 papers). A typical topic in the papers on improving performance was the use of sensors to measure performance or effort levels and provide feedback to users to improve various aspects of their skills or exercises (e.g., throwing errors in basketball, effort levels, perceived speed or golf swing performance assessment). Malachi et al. (28) choose another way of using VR in several types of physical training. They found the following applications of VR technologies in sports: as a tool for educating users, risk management in sports, sports performance analysis, and physical training.

Neumann et al. (1) emphasize another research focus that is feasible with VR tools: the study and training of sports actions with decision-making processes, which were also the subject of the review by Appelbaum & Ericson (23). The authors presented various technological options (stroboscopic training, using eye tracking and quiet eye, simulation of game situations) for supporting visual training. An increasing implementation of realistic sport-specific simulations can also be observed concerning the growth of VR simulations, particularly in the research sector. In this context, Appelbaum & Ericson (23) refer to the following sports: tennis, table tennis, billiards, archery, handball, and baseball. In the review by Richlan et al. (2023) (30), the applications are expanded to include motor and psychological skills and abilities, including perceptual and behavioral skills, strategic, tactical, and decision-making skills, response to unexpected events, and enhancing mental resilience and mental performance under pressure.

The literature review by Yuqing et al. (31) explored the use of VR in various martial arts to practice several exercise techniques and discuss various modes of visual feedback. In summary, they argue that users in VR should be able to interact with several people in a comfortable way. However, it is not always clear which cues are used to stimulate the corresponding reaction behavior. In this respect, Müller et al. (3) suggest that knowledge and experience of experts should be included in this process.

In their overview article, Harris et al. (25) point out that realistic haptic information is non-existent in most cases in current VR systems. Concerning VP, the artificial representation of egocentric distances in VR can impair visual depth perception, which in turn leads to conflicts in perceptual processing. The authors assume that the resulting conflicts also affect the execution of motor skills in VR. They further conclude that the shift from dorsal to ventral action control may lead to a fundamental discrepancy between virtual and real abilities, with important consequences for understanding perception and action in the virtual world. This means that few binocular cues for depth information, conflicting depth information, and limited haptic feedback can interfere with the specialized, efficient online control of actions that characterize the dorsal stream.

#### 4.1.2 Objective 2: requirements for the visualization of the user's body in Vr

Gerschütz et al. (24) presented approaches to achieve a realistic VP and interaction in a virtual world. The authors also demand a complete virtual body representation based on the study results of Mohler et al. (32) to achieve a higher degree of immersion and realistic distance estimation. For this, the virtual body must be an exact equivalent of the user's real body, for which high-precision 3D scans should be used. In addition, the movements of the virtual body should be carried out using a high-precision markerless tracking system, whereby interference with the user by additional devices must be avoided.

In their overview article, Harris et al. (25) discuss the problem of disembodiment in VR, which can disrupt normal action control. The inclusion of a virtual body not only leads to a stronger sense of presence but also influences distance estimation, which is fundamental for action planning. It is concluded that an inadequate representation of the physical body is a further obstacle to realistic action control in virtual scenes. Images of objects do not appear to activate action responses in motor networks of the dorsal stream like tangible real objects. For objects in the real world, the three-dimensional properties and stereo characteristics inform the viewer how to grasp them. However, the unconventional methods of interacting with objects in VR, such as using handheld controllers, can disrupt this natural interaction (25). A possible solution to this problem could be the combination with motion capture, although this is an expensive option. In the review by Putranto et al. (29), the combination of VR with motion capture to transfer the movements recorded by motion capture to an avatar (own body, trainer, or teammate) is suggested as a good tool for learning and training better strategies and tactics.

#### 4.1.3 Objective 3: transfer VR/RE

Although 10 of the 12 VR intervention studies analyzed by Richlan et al. (30) found a positive transfer of training effects to the real world in target and precision sports (e.g., archery, bowling, curling, darts, golf) and bat/racquet and ball sports (e.g., baseball, table tennis). However, there is still a lack of systematic evidence regarding the factors influencing this transfer. Therefore, future researchers should aim to identify the specific expertise levels that are relevant for increasing the probability of successful transfer to the real world. The transfer of potential training effects from the virtual to the real world can be achieved by considering two different important factors: (i) validity (i.e., the extent to which the characteristics of the virtual environment resemble the characteristics of the real world) and (ii) fidelity (i.e., the similarity between the behaviors elicited by the real world and the VR application) (30). In addition, achieving the highest possible reproduction may not be essential. In this regard, immersion (or “presence”, i.e., the feeling of being present) in the context of VR interventions should always be evaluated by the user.

Gaze behavior, as an important factor for VP in VR, should be compared with that in RE to be able to make further statements about VP in both environments. The possibilities of integrating eye-tracking systems in HMD to improve user-friendliness, interaction methods, image presentation, and VP analysis in future physical training scenarios should also be examined (7).

### 4.2 Analysis of research articles

Table 3 contains the 46 research articles based on which the objectives are to be processed. For a better overview, the aim, sports domain/application, type of sports, test subjects, VR technology used, and the key findings were noted for each publication.

**Table 3:** Research articles dealing with visual perception in VR and sport (authors in alphabetic order).

Authors	Year	Aim	Sports domain/application	Type of sports	Subjects	VR technology	Key findings
Bonfert et al. (33)	2022	Impact of foot visibility on penalty shooting in soccer	Visibility of body segments	Soccer kicking	*N* = 28 [age range: 17 …. 59 years (M = 30.1, SD = 13.5)]	HTC Vive Tracker 2.0	– Players with invisible foot needed 58% more attempts. – With foot visibility the self-reported body ownership was higher
Bürger et al. (19)	2022	Comparison in beginner training of balance beam tasks in VR with similar training in RE	Motor learning	Gymnastics	*N* = 17 (14f, 20 m), mean age: 22.8 (±2.3 SD) years	HMD (HTC Pro Eye)	– Skills adapted in VR could be transferred into RW and that the VR training was as effective as the RW training in improving the movement quality of balance beam elements
Caramenti et al. (34)	2019	Determination of whether physical activity affects perceived visual speed when running	Visual speed perception	Running on treadmill	30 healthy participants (15 f) mean age: 23.25 (±2.2 SD) years	Virtual environment projected on screen in front of the treadmill	– Designing treadmill-mediated virtual environments depends on volume and type of physical activity
Colombo et al. (35)	2022	– Definition and validation of an experimental protocol for quantitative evaluation of the effects of VR on perceived effort – Investigate whether a higher degree of immersion or interaction induces a lower perceived exertion	Effect of VR to effort	Cycling	Healthy young adults, age range: 18 … 40 years	VR system developed for COPD, HTC Wive	– Rate of perceived exertion (RPE) was not influenced by any VR factor (*p* = 0.224), although showing a tendency to decrease in the case of immersive VR (RPE = 11)
Covaci et al. (36)	2015	Impact of different visual conditions on performance	Visual feedback	Basketball free-throw simulator	20 healthy participants mean age: 27.25 (±8.23 SD) years 7 experts mean age: 25.42 (±11.08 SD) years	Simulated ball trajectory	– Guidance ellipses tended to compensate the distance underestimation effect partly and helped the users throw the ball similar to the experts’ trajectories – An incorrect perception of distance can affect velocity and acceleration.
Dhawan et al. (37)	2015	Presentation of immersive, interactive VR bowling simulator for cricket batting	Anticipation of ball trajectory	Cricket	One batter	HMD	– high level of presence allowing for a real-time egocentric view-point to be presented to participants.
Drew et al. (38)	2020	Comparison of task performance and perceptual-motor involvement in a dart-throwing task between a VR training group and a group in real-world conditions (VR vs. RW)	Motor learning of dart-throwing task	Dart throwing	*N* = 50, minimum age of 18 years	HTC Vive CPO	– VR-trained group performed significantly worse in throwing accuracy than the RW-trained group and their baseline performance. – The VR-trained group reported greater acute visual symptoms compared to the RW-trained group. – VR training impaired real-world task performance, suggesting that virtual environments may offer different learning constraints than the real world.
Finnegan et al. (39)	2023	Investigation whether manipulating an individual’s expectation of effort (determined by a virtual hill gradient) may alter their perception of breathlessness	Endurance and subjective perception of effort	Cycling	*N* = 19 (10f, median age: 20 years, range: 19–41 years)	HMD, Bicycle Power Simulator	– With the help of an immersive VR cycling environment, the perceived shortness of breath can be controlled independently of physical exertion
Geisen et al. (40)	2024	Development of an innovative real-time feedback method for golf putt learning	Motor learning with visual feedback	Golf	*N* = 25 (8f, Mean age: 25.52 ± 3.32 years)	– Extended reality – HoloLens 2 glasses	– Extended reality-based real-time feedback elicited comparable learning effects as the group with technical instructions.
Godse et al. (41)	2019	Can manipulation of visual perception of objects influence actual performance in the real world?	Sports training in golf	Golf	*N* = 39	VIVE head-mounted display and tracking system	– Putting performance increased after virtual reality training exposure when virtual objects were larger (perceived as easier to hit) and decreased when virtual objects were smaller (more difficult to hit)
Gong et al. (42)	2024	Introduce VolleyNaut—an innovative virtual reality (VR) training system	Training	Sitting volleyball	*N* = 20 (age range: 18–27 years)	VolleyNaut: simulator	– Revealed consistently positive results across all user groups (volleyball players from college teams, national sitting volleyball players, and coaches)
Harris et al. (43)	2020	exploring structural learning theories using golf putting	Motor learning	Golf putting	Experts *n* = 11, 11 m, (age: 29.2 ± 13.7 years) Novices *n* = 41, 21 m, 19f (age: 21.6 ± 1.5 years)	HMD + Eye tracking	– Underlying skill structures can be learned in VR and transferred to the real world – Perceptual deficiencies will place limits on the use of VR
Harris et al. (44)	2023	Stabilising gaze during visually-guided behaviour	Quiet eye	Golf	Novice golfers, *n* = 127	HTC-Vive+game engine+Tobii Eye tracking	– Performers persist with a long pre-shot fixation even in the absence of visual information, and the stillness of this fixation confers a functional benefit that is not merely related to improved information extraction
Hosp et al. (45)	2021	Expertise Classification of Soccer Goalkeepers in Highly Dynamic Decision Tasks	Decision making	Soccer goal-keeping	*N* = 33 (novices, immediate, experts)	HMD, 360° footage	– Deep learning approach that independently finds latent perceptual features in fixation image patches. – This model is a first step in the direction of generalizable expertise recognition based on eye movements
Hülsmann et al. (46)	2019	– assessed whether participants felt like they owned and were in control of the virtual character – exploration of whether possible differences arising in motor performance across groups could be associated with differences in body ownership and sense of agency since the perspective of view is known to affect the sense of ownership	Imitation learning/motor learning	Squart movements	35 healthy, naïve participants (21 m, mean age: M = 26.3, SD = 4.4)	low-latency cave automatic virtual environment (CAVE)	– Advantage of the groups that observed their avatar performing the squat together with the superimposed skilled performance for some of the investigated parameters, depending on perspective – it can be beneficial for novices to watch themselves together with a skilled performance during execution, and that improvement depends on the perspective chosen.
Ishibe et al. (47)	2020	Development of a system for identifying the abilities of perception involved in hand-eye coordination (HEC) as it relates to badminton smash it is considered as badminton’s most effective shot.	Hand eye coordination	Badminton, Ball games	– Not experienced ball game: *n* = 6 – Experienced ball game (excluding badminton): *n* = 6 – Experts: *n* = 6	HMD	– The ability to follow the shuttlecock with the eyes is a perceptual ability related to hand-eye coordination in badminton – Differences between experienced badminton players and other game players
Klatt et al. (48)	2019	Investigation whether possible attentional and perceptual asymmetries affect sport-specific decision-making	Football-specific decision-making task	Football	*N* = 27 athletes (M = 23.22 years, SD = 2.03 years)	2.4 × 6.0 m IGLOO dome	– No differences in accuracy rate between the left and right visual field side for stimuli presented close to the screen centre in an object-detection
Ku et al. (49)	2022	– Importance of stable posture, provide immediate feedback for inaccurate posture, visualize the overall posture and feedback on the most incorrect parts to the user, and have a clear step-by-step learning guide – Using a virtual coach	Motor learning for beginners: forehand drive	Table tennis	2 coaches and 6 senior players	HMD	Positive qualitative feedback from users
Li T. et al. (50)	2022	– Development of a virtual skiing system for harmonious human-computer interaction – Provide a response environment by controlling the head vision, hand, and lower extremities movements	Motor learning Technical device	Skiing	*N* = 1?	– Visual, acoustic, and haptic feedback – HMD	System is operable and has a certain sense of immersion
Limballe et al. (51)	2022	Novel experimental protocol to examine the effect of different levels of Gaussian blur on interception performance and eye gaze data using an immersive VR task	Anticipation	Boxing	*N* = 18 (5f, Mean age: 29 years, SD = 11 years	HMD	System has the potential for using VR to provide a controlled manipulation of the visual environment while at the same time preserving a normal perception-action coupling
Loiseau Taupin et al. (52)	2023	– Examination and comparison of the impact of different viewing conditions (e.g., 3D-360°VR and 2D video displays), on gaze behavior and head excursions in a boxing-specific anticipatory task – With each viewing mode, including the level of presence experienced	Gaze behavior	Boxing	*N* = 32 (16f)	VR headset (Pico Neo 3 Pro Eye), with two different viewing modes: 3D-360°VR and 2D.	Results demonstrate that 360°VR elicited shorter fixation durations but mostly greater head excursions and immersion than a 2D projection in the context of a boxing-specific task.
Luke et al. (53)	2022	– Examination of visual attention associated with surfing expertise – Facilitation systematic analysis of performance in uncontrollable environments	Perceptual-cognitive decision-making skills	Surfing	Experts (*n* = 12, 3 m, mean age: 22.83 SD: 7.03 years)) Novices (*n* = 12, 8 m, mean age: 29.33, SD: 8.52 years))	360° video in HMD	– surfing expertise is associated with more optimal visual attention to cues informing wave approach and wave dynamics – Experts look at these areas earlier than novices, and for more time overall. – The findings suggest the performance advantages of early planning of motor actions, along with moment-to-moment adjustments while surfing.
Lynch et al. (54)	2019	Investigating whether a representation of the center of gravity (CG) can be useful for training purposes, by using this representation alone or by combining it with the local motion cues given by body parts	– Perception – Perception action	Rugby	*N* = 16, 7 experts (male), 9 novices (male) Mean age: 24.71 ± 2.43 years	4-screen CAVE	CG as a global motion cue was not sufficient in correctly detecting the attacker’s direction. – CG distracted the participants rather than aiding them
Morice et al. (55)	2023	Theoretical and methodological considerations for optimizing the use of artificial intelligence (AI) in the development of a virtual reality setup (VR) for sports science use.	Deceptive movement in basketball simulator	Basketball-throwing	-	Combining Google’s MediaPipe SSD networks capture the movement of a real attacker attempting to score in a virtual basket, and LSTM networks classify the attacker’s intentions and “intelligently” select from a library the animation of the defender’s avatar was developed	– Device can be used by coaches to detect expertise and train attackers’ creativity in producing deceptive movements. – AI-based virtual reality offers innovative theoretical, methodological, and applied perspectives for sports sciences provided that developments are rooted in an interdisciplinary framework
Nasu et al. (56)	2024	Perception training based on the kinematics of the opponent and the flight information of the ball	– Anticipation training – Perception and action	Softball	*N* = 17f [training group: *n* = 9, age: 24.9 ± 2.9), control group (*n* = 8, age: 23.5 ± 4.0)	HMD (Meta Quest 2; Meta Platforms Inc., Menlo Park, CA, USA)	Perception training alone improved both perception and action reactions.
Oagaz et al. (57)	2021	Transfer of sports skills learned in VR to real-world	Motor learning and transfer to real-world	Table tennis	Experimental group (*n* = 8), control group (*n* = 7)	– VR table tennis system that incorporates customized physics with realistic audio-visual stimuli, haptics, and motion capture to enhance VR immersion – HTC VIVE controller that provides haptic feedback	Complex skills can be learned in VR and the obtained skills can transfer to the real-world
Okada et al. (58)	2023	Ski training system using VR that enables beginners to acquire skiing skills without going to an actual ski ground	Motor learning by technical device	Skiing	*N* = 9 (beginner)	The virtual ski resort was constructed using Unity	Subjects trained with the proposed system acquired more skiing skills than subjects who did not use the system on actual ski slopes
Pastel et al. (21)	2020	– Comparing sports-related tasks in VR and reality with further manipulations (occlusions of body parts) of the virtual body – Different body visualization parts	- Visual perception of the own body	Balance, throwing, and grasping tasks	*N* = 20 (7f, age: 21.6 ± 1.6 years)	HMD (HTC Pro Eye)	– Significant differences between reality and VR – The whole-body visualization leads to the best performances – For task completion, however, it is not always necessary to visualize the whole body – Significant loss in movement quality when no body part was visualized
Pastel et al. (21)	2022	It should be clarified whether older adults also need to visually perceive their bodies in VR scenarios to the same extent as younger people in order to increase usability	Visual perception of the own body	Balance, throwing, and grasping tasks	*N* = 42 Youg-aged group (*n* = 21, 12f, age: 23.1 ± 3.3 years) Older-aged group (*n* = 21, 13f, 63.14 ± 6.98 years)	HMD (HTC Pro Eye)	VR for the elderly with caution to the task demands, and the visualization of the body seemed less crucial for generating task completion
Pastel et al. (59)	2022	Comparison between VR training and video training to learn a karate technique	Motor learning	Karate	*N* = 83 (33f, 50 m, age: 22.92 ± 3.11 years)	HMD (HTC Pro Eye)	VR training leads to similar learning results as video training, both when the entire body is visualised and when only the forearm is visualised.
Perrin et al. (60)	2019	Estimation of the error between actual and perceived locomotor speed in VE using an enactive approach	Speed perception assessment	Walking/running	*N* = 16 (4f, mean age: 23.7 ± 3.1 years)	HMD Vive	– The perception of speed in VR is strongly individual, with some participants always overestimating and others constantly underestimating – Proposal: use of individual models as recommendations for setting up locomotion-based VR applications.
Petri et al. (61)	2017	Development of an autonomous character (AC) in karate kumite, which performs attacks against a freely moving, real athlete.	– Anticipation – response to attacks	Karate	*N* = 6 (karate experts)	HMD/CAVE	– AC seems applicable for anticipation research and training in karate kumite
Rojas Ferrer et al. (62)	2020	Development of a VR system that allows both players and coaches to measure Active Visual Exploratory Activity patterns (AVEA).	Decision making	Soccer	*N* = 10	HMD combined with eye tracking	– The system makes it possible to evaluate broad exploration activity through the HMD’s head-tracking capabilities while players make passing decisions under pressure from an opposing player. – Strong negative correlation between involvement and visual exploration performance
Romeas et al. (63)	2019	Virtual life-sized perceptual-cognitive training paradigm that combines three-dimensional multiple object tracking (3D-MOT) with motor tasks	Motor-decision making	Badminton	29 university badminton athletes (6f, Mean age M = 22.98, SD = 2.77 years)	CAVE (EON cubeTM is a 7 × 10 × 10 feet room)	Initial familiarization with the unpredictable dynamic environment may be important to improving a motor dual-tasking performance
Runswick (64)	2023	Comparision between VR and 360°video about efficacy for training, ease of use, and cost	Perceptions of presence and task workload	Cricket	*N* = 39 (international)	HMD	– In VR higher levels of realism, possibility to act, physical effort, temporal constraints, and task control – In both environments: immersive experience
Sigrist et al. (65)	2015	Investigation whether multimodal audiovisual and visuohaptic feedback are more effective for learning than visual feedback only	Motor learning	Rowing	*N* = 24 (7f, 17 m, 21–33 years, mean age 26.1 years, SD 3.0 years)	Rowing simulator, CAVE, visual ocean scenario and augmented visual feedback was programmed (Unity) and projected on three screens (4.4 m × 3.3 m)	– Audiovisual feedback fostered learning of the velocity profile significantly more than visuohaptic feedback. – Well-designed simultaneous feedback promotes learning of complex tasks, especially when the benefits of different modalities are utilized.
Soltani and Morice (66)	2023	Understand what a basketball simulator can reveal about shooting performance at different scales of analysis	Validity of simulator	Basketball shooting	*N* = 12 experienced (3f, 21.0 ± 2.7 years) *N* = 10 (5f, 30.4 ± 5.5 years)	3D simulation	Coaches can trust the basketball shooting simulator to enable realistic behavior, track between-player differences, and challenge the players’ perceptual-motor systems naturally
Tian et al. (67)	2024	Enhancing Tai Chi Training System: Towards Group-Based and Hyper-Realistic Training Experiences	Hyper-realistic multi-user action training platform	Tai Chi	*N* = 39 (17f) mean age: M = 20.22, SD = 1.57 years	HMD, network intercon-nection for fewer than 20 VR devices (MR)	– VR performs the best in training accuracy, but MR provides superior social experience and relatively high accuracy. – Compared with VR, MR provides more realistic avatar companions and a safer environment.
Tian et al. (68)	2023	– Proposal: Kung Fu Metaverse: A new movement guidance training system	Movement Guidance Training System (teaching and learning)	Kung Fu	*N* = 17	VR + AR = mixed Reality (MR) Unity was integrated and packaged for transmission to HMD. Binocular resolution of 4,320 × 2,160, a field view of 105°, and a refresh rate of about 72 Hz (up to 90 Hz).	– System allows free movement and significantly reduces adverse effects such as sickness and dizziness. – The addition of the composite navigation module improves movement precision during practice.
Tsai et al. (69)	2021	Feasibility Study on Using AI and VR for Decision-Making Training of Basketball Players	Decision making	Basketball	*N* = 45 (9f, M = 21.12, SD = 1.89 years)	HMD and a motion capture suit are trained by intuitively interacting with the VR system and receive decision suggestions when a bad one is made	– System uses the IMU sensor to recognize the action of the trainee and feedback on whether the recognized action is a proper decision concerning the given defensive scenario – no differences in knowledge, but decision time was significantly lower than in in commercial group
Valkanidis et al. (70)	2020	Investigation of the effect of the visual occlusion of the initial ball trajectory by the wall on the performance of naïve participants and skilled goalkeepers.	Effect of the visual occlusion of the initial ball trajectory by the wall on the performance of naïve participants and skilled goalkeepers	Football goalkeeper	Novices: *N* = 15 ((age 27.9 ± 6.2 years) Skilled: *N* = 10 (age 22.4 ± 3.7 years)	HTC Wive	Results cannot suggest an all-out removal of the wall–this study only considered one potential downside–but should motivate goalkeepers to continuously evaluate whether placing a wall is their best option.
Vargas González et al. (71)	2017	– Augmented Reality (AR) in training sports rule comprehension – Comparison between desktop, VR, and AR interfaces	Learning of rules and regulations	Soccer	*N* = 36 (13f, mean age: 23.1 years, median: 21)	HTC Vive	No significance difference among VR and AR; however, these participants outperformed the Desktop group which needed a higher number of adaptations to acquire the same knowledge
Waltemate et al. (72)	2016	Impact of feedback delays on perception and motor performance as well as on their interplay in virtual environments employing full-body avatars	Visual feedback	Participants were instructed to simultaneously mimic the movements of the ghost character	Ten naıve participants (4 m, mean age M = 23.2, SD = 2.2, 9 right handed)	– CAVE (Lshape, 3 m × 2.3 m for each side). – This mirror showed a reflection of the room itself as well as a virtual avatar.	– Motor performance and simultaneity perception are affected by latencies above 75 ms. – Although sense of agency and body ownership only decline at a latency higher than 125 ms, and deteriorate for a latency greater than 300 ms, they do not break down completely even at the highest tested delay.
Weng et al. (73)	2022	VR learning and web-based feedback system designing an intuitive system for efficient communication between the trainee and the coach	Motor learning	Table tennis	*N* = 8 (4f, aged between 21 and 27)	– Participant wearing IMU suit for motion capture – Trainees can examine their mistakes through a tablet or an immersive VR world.	Trainees could transfer the movements learned in our system to real-world training

#### 4.2.1 Objective 1: sports domains and sports disciplines

Looking at the various sports areas, the following applications can be found in the sports disciplines (multiple answers are possible), which in particular also describe the visualization methods used in VR (Table 4):

**Table 4:** Systematization of the research articles found according to sports domains and sports types.

Sports domain	Review
Contribution to improving health: football, basketball, table tennis	Putranto et al. (2023) (29)
Improvement of sport-specific skills	Putranto et al. (2023) (29) Richlan et al. (2023) (30)
Training of strategies and tactics in-game sports	Putranto et al. (2023) (29)
Education	Malachi et al. (2023) (28)
Risk management in sports	Malachi et al. (2023) (28)
Sports performance analysis	Malachi et al. (2023) (28)
Physical training	Malachi et al. (2023) (28) Hoang et al. (2023) (26)
Training of strategy and tactic	Richlan et al. (2023) (30)
Decision-making skills	Richlan et al. (2023) (30)
Improvement of mental performance	Richlan et al. (2023) (30)
Motivation	Hoang et al. (2023) (26)
Engagement	Hoang et al. (2023) (26)
Anticipation skills	Müller et al. (2023) (3) Yuqing et al. (2021) (31)

The results for the stated sports domains are summarised and discussed in the following.

#### 4.2.2 Motor learning

Most research papers were found for different sports in motor learning with a focus on VP. VR is particularly used for learning relatively simple movement techniques, such as the snow plow (58). A wooden base is used in this study to support the learning of the correct posture, which provides the test person with additional haptic feedback for foot control. Another way to obtain feedback on posture and movement control is to use an inertial measurement unit system that measures the user's posture and movement in real time and provides corresponding feedback (50). To increase the degree of immersion, both studies display snowy landscapes as virtual environments.

Weng et al. (73) use step-by-step instructions for learning movement techniques in table tennis, which are represented graphically using numbering and arrows. Furthermore, a remote trainer provided web-based feedback. The participants were able to prove that this visual support is helpful when learning movement sequences and that a transfer to reality is possible.

Another way of using VR in the learning process is the visualization of the ball trajectory in table tennis (57). Moreover, a tracker was mounted on the paddle to create as realistic a feeling for the stroke movement.

Adequate feedback as quickly as possible is particularly important in the motor learning process (73). Another variant of visual feedback is presented by Sigrist et al. (65) for rowing. The virtual oar is displayed alongside the real oar, with the transparency of the target oar indicating the degree of deviation.

Drew et al. (38) show that dart-throwing training in VR leads to poorer accuracy than under real conditions a possible reason for this could be that VR training platforms do not reflect the same vividness and immersion as real environments, which impairs kinematic movement patterns. To improve the realistic impression, acoustic signals are also increasingly being used, such as when the golf ball makes contact with the club (43). However, this extended feedback is less efficient for learning simple motor tasks than for complex tasks (43). Here, Sigrist et al. (65). show for rowing that audiovisual feedback promotes the learning of the speed profile more than visual-haptic feedback. It can currently be observed that VR is also increasingly being combined with augmented realities (68). For example, the extended reality method is also used, such as the inclusion of Hololens (mixed reality glasses that allow the user to display interactive 3D projections in the immediate environment with the support of a natural user interface) in the real environment. Geisen et al. (40) use this method for golf putting by applying a virtual golf club in reality.

In conclusion, the following aspects of learning are covered in the studies:

• Learning of relatively uncomplicated movements (46, 49, 58),
• Learning complex movements at a similar level to video technology (38, 68, 74),
• Graphic support through additional information (46, 57, 65, 73),
• VR in combination with AR (40, 68),
• Use of additional non-visual aids (43, 50, 58).

#### 4.2.3 Visual perception and its manipulation

To be able to assess the presence in VR, it is often common to compare similar situations between a VR environment and a 360° video. In this regard, Runswick et al. (64) compared the invasive experiences of playing cricket in VR with those in a 360° video montage. Participants reported that they had an immersive experience with both methods, but with VR they had a higher level of realism, agency, physical exertion, time constraints, and task control. In contrast, 360° videos offered better opportunities to perceive the surroundings in visual detail.

VP and its controlled manipulation for sport-specific training goals play a special role in sports movements in VR. The 9 papers found are related to the visualization of one's own body (cf. objective 2), perceived speed, the manipulation of visual conditions, visual feedback, perception-action interaction, VP of the opponent, and the perception of presence.

VR can, for example, provide visual feedback when running on the treadmill, reducing monotony and increasing motivation. However, the relevance of using VR tools for locomotion depends on the ability of these systems to provide a natural, immersive feeling, especially a consistent perception of speed. The aim of the studies (60) and. (2019) (34) was to evaluate the discrepancy between actual and perceived locomotion speed in VR with active control of the VR environment. The results show that the underestimation of both speed levels (8 km/h and 12 km/h) in VR varies from individual to individual and depends on the level of experience. The percentage underestimation for sedentary was up to 35% of the actual set treadmill speed. Physical fitness, however, proved to be a poor predictor of visual speed perception. To increase user engagement and participation in exercise programs, the development of “personalized” virtual environments on the treadmill practitioners should take into account the personal characteristics of users to provide the most natural and engaging feedback possible.

With earlier VR technologies (large-screen immersive display environments such as Cave Automatic Virtual Environment or CAVE) distances were often underestimated (36). This leads, for example, to the basket being hit less often in basketball free-throws. The authors therefore work with graphic guide ellipses that partially compensate for the effect of underestimating the distance and allow users to throw the ball in a similar way to the experts.

#### 4.2.4 Decision-making process

As the 6 papers researched in soccer goalkeeping, football or soccer, surfing, badminton, and basketball show, decision-making processes can be specifically analyzed and trained in VR. In the study by Luke et al. (53), visual attention was examined in connection with motor skills. Experienced and inexperienced surfers watched 360°surfing videos using an HMD. Eye tracking, presence, and engagement were measured. The results show that surfing experience is associated with more optimal visual attention to cues of wave approach and wave dynamics. The results indicate the performance benefits of planning motor actions early and making moment-to-moment adjustments during surfing. In other sports such as basketball, decision-making plays a crucial role when the offensive player has to make effective decisions in response to different defense behavior by opponents in order to score a basket. A respective study by Tsai et al. (69) shows that the VR system with computer-simulated content has a positive effect on decision times in comparison with a 360° panoramic video. Romeas et al. (63) present a virtual life-size perceptual-cognitive training tool for badminton, which enables motor and perceptual sports decision-making tasks. The number of shared attentional resources in the type of additional task (i.e., perceptual or motor decision-making) appears to be key to interpreting dual-task interference and should be considered when designing representative multitasking training for perception and cognition.

To achieve a high presence of whole-body interactions, Rojas Ferrer et al. (62) propose a combination of an HMD and kinetic body tracking. This was proven to be a great advantage for football decision-making processes. Using an immersive simulated, ecologically valid football task, Klatt et al. (2020) investigated whether possible attentional and perceptual asymmetries influence sport-specific decision-making (48). No performance differences in accuracy were found between the left and right sides of the visual field for stimuli presented near the center of the screen in an object recognition (perceptual-based) and feature recognition (attentional-based) task. However, accuracy was higher when the stimuli were presented at an angle far from the center of the screen, on the left side than on the right side of the visual field. Thus, it can be concluded that corresponding VR technologies offer opportunities to investigate new perspectives on attentional and perceptual asymmetries in real-life scenarios.

Decision-making is also significantly influenced by expertise in the relevant sport. Nevertheless, there are no consistent statements about specific perceptual characteristics that explain expertise. Hosp et al. (45) present a neuronal network based model for the assessment of expertise in goalkeeper-specific decision-making tasks in build-up situations. A deep learning approach was developed that independently finds latent perceptual features in fixation image fields. The average accuracy of this prediction model is approximately 73%.

#### 4.2.5 Anticipation skills

VR tools can also be used, as the following studies show, to train anticipation skills, which are essential for success in many games and martial arts. The four papers found deal with boxing, softball, karate, and cricket. Petri et al. (61) developed a nearly autonomous character in karate kumite, which performs attacks against a freely moving, real athlete. Based on the athlete's position and distance, the virtual opponent performs an appropriate karate attack. The athlete should react to this as quickly and efficiently as possible. Based on a survey of experts, this autonomous character appears to be suitable for anticipation research and training in karate kumite.

Video playback has been used extensively in the past to study anticipatory behavior. But in two-dimensional videos, the VP for necessary sporting reactions is impaired (37). The authors present a cricket simulator as a combination of motion capture and VR, whereby various cricket scenarios are presented to the participant in an egocentric view. Users reported positive experiences when using the system.

Limballe et al. (51) use a visual blur method to focus visual behavior on relevant stimuli for anticipating the opponent's attacks in boxing. The study demonstrates the use of VR as a means of visually manipulating the environment, which has the potential to support learning and transfer to real-life sporting situations. Nasu et al. (56) used VR anticipation training for softball by visualizing not only the opponent's posture and movement but also the ball trajectory.

#### 4.2.6 Training

VR technologies are particularly effective at providing users with visual feedback, making them valuable tools for training purposes. A number of studies show how VR tools and their visualization options are used in training in the sports of alpine skiing, basketball, golf, tai chi, and sitting volleyball. However, to design efficient VR training tools, it is important to know what needs to be considered in VR.

In their study on golf, Godse et al. (41) found that hitting performance in RE increased after training in VR when virtual objects were larger (perceived as easier to hit) and decreased when the virtual objects were smaller (more difficult to hit).

Li et al. (75) present a multidimensional ski training system. The translation platform with 6 degrees of freedom allows the realization of a wide range of slalom training and many terrain simulations. This is achieved through specific algorithms, improving the alpine skiing experience and making it easier for users to train on different terrains. To visualize the speed of the environment, algorithms are used that simultaneously incorporate the speeds of the skies and the controllers, giving the user a realistic impression.

VR training systems are also used in para-sports. VolleyNaut (42) is a specialized VR training system designed to address the limited availability of sports facilities for sitting volleyball, effectively filling a crucial gap in the training resources for this para-sport. The user can train daily and special scenarios are available, with the emphasis on realistic rallies. The system uses input data to determine the position of the player, the location of the supporting machine, the type of ball provided, and the appropriate performance evaluation model for each task. The variability of the scenarios and ball speed suits beginners and experts alike. VR also makes it possible to collect extensive data, including the trajectory of the ball, landing point, and contact points of the ball, which is essential for analyzing the sitting volleyball and improves the effectiveness of this VR training tool.

The study by Tian et al. (67) on Tai Chi training shows another application of VR tools. The system comprises several independent VR devices, creating a realistic multi-user action training platform. At the same time, users are provided with real-time visualized hand movement trajectories for action guidance, resulting in rapid improvements in both movement precision and social experience.

Valkanidis et al. (70) looked at the defensive wall in soccer and its relevance for the goalkeeper to prevent a goal from a free kick. VR was used to investigate how the visual occlusion of the initial trajectory of the ball through the wall affects the performance of less and better experienced goalkeepers. As a result, it was shown that the wall built up by teammates influences the view of early movement sequences at free kicks with an arc-shaped ball trajectory, which can impair the goalkeeper's performance. This seems particularly relevant when there are experienced free-kick takers for whom the wall is not a blockade and less experiences goal keepers need experience with this.

#### 4.2.7 Other sports domains and applications

A few studies with other sports domains and applications will be discussed below. Firstly, a study by Colombo et al. (35) should be mentioned here, which investigates the extent to which VR influences the perception of stress in the area of rehabilitation when cycling. As a result, it was found that the level of perceived effort was not significantly influenced by any VR factor (degree of immersion or interaction), although it tended to decrease with immersive VR. A discrepancy between afferent signals and expectations may underlie unexplained respiratory distress.

Using a novel immersive VR exercise paradigm, the study by Finnegan et al. (39) investigated whether a virtual slope manipulation can change the individual's expectation of exertion and thereby alter the perception of breathlessness, independent of actual physical exertion during cycling. It has been shown that the individual's expectation of the effort resulting from the different slopes of the cycle route is just as important for the perception of breathlessness as the actual effort required to cycle.

In badminton, the ability to follow the shuttlecock with the eyes is a perceptual skill related to hand-eye coordination (HEC). The aim of the study by Ishibe et al. (47) was to develop a system to identify the perceptual skills involved in HEC when it comes to the badminton smash, which is considered the most effective stroke in badminton. The two VR systems developed to identify the perceptual abilities of the HEC can help evaluate and train perceptual ability during smash receptions.

To investigate the quiet eye effect in golf in VR, Harris et al. (44) used an HMD with built-in eye tracking. The results showed that performers persist with a long pre-shot fixation even in the absence of visual information, and the stillness of this fixation confers a functional benefit that is not merely related to improved information extraction.

When virtual simulators are used in sports training, their validity must be checked beforehand. Soltani and Morice (66) demonstrate this for a basketball shooting simulator with positive results. Coaches can therefore be confident that the basketball shooting simulator provides realistic behavior, highlights differences between players, and challenges the players' perception and movement systems in a natural way. The similar device by Morice et al. (55) can be used by coaches to detect expertise and train attackers' creativity in producing deceptive movements. The AI-based virtual reality VR offers innovative theoretical, methodological, and applied perspectives for sports sciences provided that developments are rooted in an interdisciplinary framework (55).

#### 4.2.8 Objective 2: requirements for the visualization of the user's body in VR

The visualization of the user's body and that of the trainer plays a decisive role in sports training. This was realized in table tennis for learning the basic stances and the forehand counter (49) and the correct execution of squats (46). In a virtual mirror, users could compare their movement or posture with that of the coach and correct it. The visualization was realized with a motion capture system. Pastel et al. (22) also used a virtual mirror and a virtual trainer to learn a complex karate technique. The learning success was similar to that achieved using a video technique.

The VP of the opponent also plays an important role in team sports and martial arts. Lynch et al. (54) focused on the reaction in rugby using a 4-screen CAVE. Within their study, experts were more successful in identifying the future passing direction of an opponent than novices. These findings were consistent for both perception and perception-action tasks.

Since the visualization of the entire body, especially the user's own body, can lead to latencies and thus to an impairment of the perception of presence due to the high computing effort, the question arises as to whether the quality of the movement deteriorates if the user cannot see his entire body. In the study by Pastel et al. (21), the influence of VP of one's own body on performance in three motor tasks (balancing on a beam, grasping a ball and placing it on a target area, and throwing a ball into a goal) was investigated. The results demonstrate that it is not necessary to visualize the entire body in VR to complete the motor tasks without any considerable loss of quality. It was found that the older test subjects were less influenced by the body visualization than the younger ones. It can be assumed that younger adults react more sensitively to the body visualization than older individuals (59). The authors recommend visualizing at least one body part, as performance is significantly worse if no body part is visualized. It can be concluded that only the body segments that are relevant to the movement and that need to be seen by the athlete to perform the movement task have to be visualized. Pastel et Al (59). were able to show that it is even possible to learn a whole-body movement by visualizing the forearm alone. Waltemate et al. (2016) investigated the influence of the latency of the virtual mirror image of one's own whole body on motor performance in a CAVE (72). The feeling of sense of action and body ownership deteriorates with a latency of more than 75 ms to 125 ms. However, participants reported that the cause of this was seen more in their motor errors than in the actual degree of delay (72).

Lynch et al. (54) investigated whether a representation of the center of gravity is sufficient and useful for training purposes in rugby. As a result, it was found that the visualization of the body's center of gravity as a movement cue is not sufficient to correctly identify the direction of the attacker. The visualization of the center of gravity distracted the participants rather than supporting them (54).

The fact that at least individual body parts should be visible becomes clear in analogy to (21) and (33). Bonfert et al. (33) investigated how the visibility of the foot influences the penalty shooting of inexperienced to advanced soccer players. When the players saw the visualized foot, the accuracy of the shots and self-reported body control were significantly better.

In softball, Nasu et al. (56) combined the visualization of the opponent's kinematics with the flight information of the ball to train the visual system required for real-life situations. It was shown that VP training in VR in softball leads to improvements in motor reaction ability and discrimination of ball speed. This study showed the effectiveness of pure perceptual training and emphasized the need for thoughtful use and design of this training.

#### 4.2.9 Objective 3: transfer VR/RE

The findings of Harris et al. (43) show the challenges and opportunities of VR as a training tool and emphasize the need to empirically test the costs and benefits of specific systems before VR training is used. This also includes transferring the training performance in VR to the conditions in RE. Based on the study in golf, Harris et al. (39) conclude that skill structures can be learned in VR and transferred to RE [Oagaz et al. (57) and Weng et al. (73) demonstrated this in table tennis for athletes at beginner and intermediate skill levels. Oagaz et al. (57)]. conducted posture and movement training in table tennis with feedback through body angle visualizations as well as ball return training The findings support the notion that complex skills can be learned in VR and that obtained skills can transfer to the real world. The authors offer a low-cost VR table tennis training platform that provides effective training through real-time feedback on motor skills and ball return. However, the performances are not always compared with a group that has trained in reality (58), which means that statements on the transfer from VR to RE lose valuable aspects. However, the accuracy of movement seems to be worse with a VR learning or training tool than under real conditions. However, the use of simulation possibilities in VR training can, under particular conditions, lead to training results similar training results as to those in the real world. This is shown in the study by Bürger et al. (19), in which beginners who perform gymnastics elements on a beam with simulated height in VR achieve similar learning progress to training in the real world.

## 5 Discussion

Based on the assumption that visual perception is crucial for athletic performance and that VR tools are becoming increasingly important in sports, the role of visual perception and its use in VR in sports was investigated. Different aspects were considered, which resulted in three research objectives. The corresponding findings will be summarised in this concluding discussion.

### 5.1 Objective 1: sports domains and sports disciplines

Following Neumann et al. (1), who found that most VR applications relate to cycling, rowing, weightlifting, and golf, they demand that VR technologies should also be used in skill-based sports. The learning and training of sport-specific movement techniques and the training of decision-making processes are seen as particular areas of application. Table 5 shows the allocation of the review articles to the various sports domains.

**Table 5:** Assignment of reviews to the sports domains.

Sports domain	Number of papers	Type of sports
Main sports domains		
Motor learning	13	Table tennis, rowing, dart throwing, Kung Fu, golf, squats, skiing, karate, gymnastics
Visual perception and its manipulation	9	Basketball, rugby, football/soccer, several motor tasks, walking/running, cycling, cricket, soccer kicking
Decision making	6	Soccer goalkeeping, football/soccer, surfing, badminton, basketball
Anticipation	4	Boxing, softball, karate, cricket
Training (general)	5	Basketball, skiing, golf, sitting volleyball, Tai Chi
Other sports domains		
General perception/feedback	2	Rowing, cycling
Hand-eye coordination	2	Badminton, ball games
Gaze behavior	1	Boxing
Learning of rules and regulations	1	Football
Quiet eye	1	Golf
Simulator evaluation	1	Basketball shooting

In the analysis of research articles, we found 46 papers that deal with the following areas, focussing in particular on visual perception in VR: Motor learning, visual perception and its manipulation, decision-making process, training, and anticipation.

### 5.2 Motor learning

Most papers were found related to motor learning. Very different types of sports (table tennis, rowing, dart throwing, Kung Fu, golf, squats, skiing, karate, gymnastics) were considered. In VR, visualizations are used in particular to improve the immersion of the user so that they move in the virtual environment as realistically as possible.

For visual feedback on body posture and movement motion capture systems (e.g., inertial measurement units (75) can be integrated into VR systems. The VR table tennis training system also offers the option of feedback by helping the coach to clearly communicate a trainee's mistakes (57).

To learn movement techniques in a virtual environment, graphic aids can be used in the form of numbering the component movements with corresponding arrows (73).

Another aspect is the combination of VR and AR technologies, which on the one hand allows real-time feedback (68) and on the other hand also enables the visualization of the sports equipment used, such as a golf club (40). Finally, it should be mentioned that the learning of relatively basic movements is well documented. Studies also show that complex movements can also be learned at beginner level (19, 59). Visual manipulations in VR, such as varying the speed of the trainer demonstrating the technique, viewing the movement from different perspectives, using a virtual mirror for user's movement and that of the trainer, visually focussing on the essential sub-movements, text, and graphic aids, all help here.

It should also be added, that acoustic signals can be used on the one hand to increase the degree of immersion, but on the other hand, they should rather be used for simple movements (43, 65).

Finally, the study by Vargas González et al. (71) should be briefly mentioned. Here it is shown that learning game rules in football is more efficient with VR or AR than with a conventional desktop. VR tools can therefore also support the learning of theoretical knowledge in sport.

Based on the studies found, it can be concluded that VR can be used in motor learning at the beginner level specifically for learning specific movement techniques with visual aids, using visual feedback on posture and movement, using a virtual trainer for repetitive practice and three-dimensional visualization of one's movement at different speeds and perspectives. The integration of acoustic and haptic signals can also support the learning effect in VR.

### 5.3 Visual perception and its manipulation

In the domain of VP, or perception-action interaction we found relevant papers with studies in games, cycling, running and walking, several motor tasks, and cricket.

To investigate VP in sport-specific situations in VR, a comparison was made with a 360° video presentation in boxing (52), surfing (53), and cricket (64). From the results, it can be concluded that a higher degree of immersion is achieved with VR, but that 360° videos show visual details of the environment better. The extent to which this has an influence on athletic performance has not been proven. More visual details mean even higher resolution for future technological developments in VR, although there is a risk of increasing latency times and thus impairing the user's interaction with the virtual world.

The perceived speed is a crucial factor that influences the degree of immersion for locomotor movements. From the results of the studies by Perrin et al. (60) and Caramenti et al. (34), it can be concluded that there is a difference between the perceived speed in VR and the real speed, especially for beginners with little sport-specific expertise. However, the underestimation of distances is no longer a problem with modern HMDs and therefore does not need to be taken into account when developing VR tools.

In return games, it seems obvious to visualize information on the ball flight and the opponent's kinematics. In this regard, the study by Nasu et al. (56). in softball showed that perceptual training alone improved both perception and reactions in the real world.

Focussing on visual perception, it can be concluded that VR achieves a higher level of immersiveness than 360° videos, although the resolution does not yet correspond to the video technology. Nevertheless, it can be assumed that VR only slightly impairs VP and can be used for perception training.

### 5.4 Decision-making process

The review by Richlan et al. (30) already emphasizes that training decision-making processes are an important application of VR. Visual attention plays an important role here. This can be systematically analyzed in VR and combined with an eye-tracking system. For example, Luke et al. (53) show for surfing that experts look at the waves earlier and longer and thus plan their motor reactions earlier and more precisely. This means that sport-specific VR simulators can also support attention training by focussing on the essential cue. Using a combination of HMD, eye tracking, and a neural network, the assessment of expertise in goalkeeper-specific decision-making tasks could be assigned to beginners, advanced, and experts with high accuracy (45). However, in order to use gaze behavior for decision-making processes, further studies are needed to compare gaze behavior in VR and in the real world (20).

Romeas et al. (63) show that training perception and decision-making processes is important for dual-task tasks and multitasking. To realize whole-body interactions to analyze responses to the virtual stimuli, such as in decision-making processes in football, the HMD should be combined with kinetic body tracking (62). VR technologies also provide further opportunities to analyze possible asymmetries in attention and perception in a sport-specific way. However, the study by Klatt et al. (48) shows that such asymmetries do not influence football-specific decision-making processes.

In summary, it should be noted that the eye tracking method can be used for decision making processes. Modern HMDs have this capability, so future studies should investigate the extent to which gaze behavior during decision-making processes in VR corresponds to those in RE.

### 5.5 Anticipation skills

VR tools are also increasingly being used to train anticipation skills. These are important in martial arts, for example, where the athlete learns which attack to launch from the posture and movement of the opponent. Only by recognizing the attack at an early stage it is possible for the athlete to prevent the attack or counter it in time (61). Until now, only simple avatars could be used in training, which are not fully autonomous. The use of artificial intelligence could contribute to the development of autonomous characters in the future. VR now makes it possible to use blur technology to mask the visual stimuli of non-essential cues, so that the athlete's attention is directed to the relevant cues (51).

VR also offers the advantage of visualizing ball trajectories so that the athlete can anticipate the flight of the ball in game sports (56). To train the ability to react and anticipate in ball sports, the occlusion method can be used in VR by masking the ball trajectory in time (70).

The following conclusion can be drawn from these findings. Currently, anticipation training is particularly useful in rebounding games and other ball sports when it comes to anticipating the trajectory of the ball and reacting accordingly. Anticipating an opponent's movements in martial arts, for example, requires autonomous avatars for optimal training, which will only be available in the future.

### 5.6 Training

This section will explain the extent to which VP is taken into account in the development of general VR training tools. One way to achieve training effects is through visual manipulation, as shown in the golf study by Godse et al. (41), in which the virtual ball was enlarged to simplify the task. The multidimensional ski training system by Li et al. (75) demonstrates how different terrain simulations can be used to design slalom training that is close to reality and rich in variety. The speed of the environment was calculated simultaneously in order to achieve the most realistic impression possible. There are reports of simulators that also enable athletes in para-sports to train daily. For example, the volleyball simulator VolleyNaut (42) enables seated athletes to use a wide variety of scenarios and ball speeds at beginner and advanced levels. Group training together with the trainer enables a virtual hyper-realistic multi-user action training platform, as presented by Tian et al. (67) for the learning and training of Tai Chi. In addition to the overlay of movement trajectories, the social aspect is also taken into account by the group training system composed of multiple standalone VR devices. An important field of application for VR training tools in endurance training and rehabilitation could be to manipulate the perceived effort by designing the virtual environment (e.g., gradient of the terrain when cycling). However, the studies by Colombo et al. (35) and Finegramm et al. (39) cannot provide clear evidence of this.

Hand eye coordination (HEC) is important for an effective return game. The results of the study by Ishibe et al. (47) can used to evaluate the ability to perceive when receiving the ball with the help of an HMD and coupled eye tracking as well as the tracking of the racket and its realistic visualization in VR.

The findings for the quiet eye in golf by Harris et al. (44), contribute to a new theoretical understanding of the quiet eye and should encourage further investigations in the future to clarify the exact functional role of stabilizing gaze in visually controlled behavior. This could then be trained specifically in a VR environment and also controlled using eye tracking.

The following VR options for application in sports training can be specified from the researched studies: visual manipulation, systematic variation of environmental conditions, multi-user action training, and using eye tracking to improve the gaze behavior.

### 5.7 Objective 2: requirements for the visualization of the user's body in VR

Some authors (33, 59, 72) investigated the extent to which the entire body of the user or other participants is necessary to achieve sufficient immersion in VR. This depends on the one hand on the specific sporting movement and on the other hand on the motor task. Visualization of the whole body is important for learning full-body movements. The visualization of one's own body and that of the trainer in a virtual mirror seems particularly promising for the motor learning process. This allows the trainee to learn the corresponding movement from different perspectives and at different movement speeds according to their needs (49). For movements that have already been learned, only visualizations of the body segments relevant to the movement are necessary.

Pastel et al. (21, 59) also achieved good results in performance of several motor tasks without visualization of the whole body. Different visualizations of their own body were not recognized by elderly adults. However, in most cases, this requires a motion capture system, which would significantly increase the effort involved in the normal training process. A possible alternative in practice would be to use fewer trackers and to model the body segments using the inverse dynamics method. Furthermore, the latency times that occur, especially with fast movements, can lead to the user not feeling fully immersed in the virtual environment.

### 5.8 Objective 3: transfer VR/RE

In their narrative review, Richlan et al. (30) reported that VR training tools have the potential to improve sports performance in reality. This can be achieved in particular by training of motor and psychological skills and abilities of athletes, including perceptual and behavioral skills, strategic, tactical, and decision-making decision making, reaction to unexpected events, and improving mental resilience and mental performance under pressure. It is also criticized that the majority of studies published only demonstrate positive effects, which creates a distorted impression of the transfer of training effects in VR to reality. In addition, future research should be conducted to determine whether different VR systems or even mixed reality and AR systems can be used for training with a clear transfer effect. Little is known about effect-dose relationships when using VR training tools.

The research articles analyzed confirm that the use of VR learning or training tools only makes sense if the transfer to the real world is possible.

Summarising the results of the studies that deal with the transfer of VR training to reality and are labeled accordingly in Table 2, the following conclusions can be drawn.

Until now, VR training has not achieved the desired accuracy of movement, which means that movement execution in the RE can even be expected to be impaired (14). One promising area for using VR in training is perception training (56). In general, VR should not only reproduce reality as identically as possible but also utilize additional possibilities. For example, ball trajectories can be simulated and other information can be presented to the user in games (56, 57). Decision-making processes can be trained by utilizing the interaction between the athlete and the VR system. For basketball, the study by Tsai et al. (69) showed that shorter decision times can be achieved with such a system than with conventional training. Feedback from the coach is essential for the learning and training process through additional assistance, sensors worn by the athlete, and a web-based feedback module for the trainer (73).

The statement by Richlan et al. (30) can therefore be endorsed that due to the small number of studies in very different sports, little is known about transfer possibilities and the design of effective VR training. Preliminary possibilities include mixed anticipation training (RE and VR) for karate kumite (15) and the learning of gymnastic elements on the balance beam with simulated height (19). It has been shown that special VR training, such as for training decision-making processes or anticipation, is promising as supplementary training, but movement learning in particular does not tend to go beyond the level of gross coordination. It is therefore perhaps a good option for beginners but less suitable for competitive sports.

### 5.9 Limitations of the review

Finally, the limitations of the review process will be discussed. Despite the inclusion of 5 databases and the search by the authors and not by automated tools, it cannot be guaranteed that all relevant studies were found. It is also quite possible that not all relevant studies were included in the databases. Another limitation could be that the search term “athletes” was not used. It is possible that some articles could still be found in this way for special sports in the competitive sports sector.

## 6 Conclusion and outlook

VR is increasingly being used in sports training. In order to utilize VR tools even more efficiently for sports training, further developments are required with regard to the technologies and programming of the tools. However, as it is not yet possible to implement all sensory stimuli in VR, there will be differences in the complex perception in VR compared to the real setting. As visual stimuli play a special role in athletic training, the current literature review aimed to provide an overview of previous studies on visual VP in VR tools with regard to three objectives: sports domains and sports disciplines, body visualization, and training transfer effects to real world. This review included 12 literature reviews and 46 research articles.

With respect to the first objective, it can be concluded that VR has been used for a variety of sports disciplines and sports domains as well as different training modalities have been realized. It can be noted that motor learning particularly benefits from the possibilities of VR, but more so for beginners. VR tools offer the learner additional aids that are difficult to realize in a real setting. However, it must be mentioned that in the current state of VR technology, VR skill training does not appear to be suitable for elite athletes. In the future, the integration of acoustic and haptic signals is expected to support the learning process in VR.

In context with the second objective, it should be emphasized how important it is that the necessary visualizations of one's own body and those of the other players are adapted to the respective sports situation. In most cases, it is not necessary to visualize your own body completely, but it is necessary to visualize the body segments relevant to movement. It is to be expected that with the help of inverse kinematics, the current lack of body visualization can be solved.

The third objective deals with the very important aspect of the effect of VR training on reality. Due to the few and inconsistent studies, it is difficult to make general conclusions about the transfer effect from training in VR to effects in reality. More studies will be needed in the future. In particular, the involvement of eye tracking in VR and RE should be used to identify important visual components for sports training. The repeatedly cited lack of haptic feedback to improve inversion in VR can be solved by incorporating the real sports equipment and its visualization visualization or through coupling with augmented reality.

## Data Availability

The original contributions presented in the study are included in the article/Supplementary Material, further inquiries can be directed to the corresponding author.

## References

[1] Neumann DL Moffitt RL Thomas PR Loveday K Watling DP Lombard CL A systematic review of the application of interactive virtual reality to sport. Virtual Real. (2018) 22(3):183–98. 10.1007/s10055-017-0320-5

[2] Duan H. Application of virtual reality technology based on computer vision in exercise systems. 2023 2nd International Conference on Artificial Intelligence and Computer Information Technology (AICIT). IEEE (2023). p. 1–6

[3] Müller S Dekker E Morris-Binelli K Piggott B Hoyne G Christensen W Attributes of expert anticipation should inform the design of virtual reality simulators to accelerate learning and transfer of skill. Sports Med. (2023) 53(2):301–9. 10.1007/s40279-022-01735-7 35881309 PMC9877049

[4] Hollman JH Brey RH Bang TJ Kaufman KR. Does walking in a virtual environment induce unstable gait? An examination of vertical ground reaction forces. Gait Posture. (2007) 26(2):289–94. 10.1016/j.gaitpost.2006.09.075 17056258

[5] Wu E Piekenbrock M Nakumura T Koike H. SPinpong—virtual reality table tennis skill acquisition using visual, haptic and temporal cues. IEEE Trans Vis Comput Graph. (2021) 27(5):2566–76. 10.1109/TVCG.2021.3067761 33750699

[6] Sauer Y Sipatchin A Wahl S García García M. Assessment of consumer VR-headsets’ objective and subjective field of view (FoV) and its feasibility for visual field testing. Virtual Real. (2022) 26(3):1089–101. 10.1007/s10055-021-00619-x

[7] Pastel S Marlok J Bandow N Witte K. Application of eye-tracking systems integrated into immersive virtual reality and possible transfer to the sports sector—a systematic review. Multimed Tools Appl. (2023) 82(3):4181–208. 10.1007/s11042-022-13474-y

[8] Dörner R Broll W Grimm P Jung B. Virtual und Augmented Reality (VR/AR). Berlin, Heidelberg: Springer Berlin Heidelberg; (2019).

[9] Renner RS Velichkovsky BM Helmert JR. The perception of egocentric distances in virtual environments—a review. ACM Comput. Surv. (2013) 46(2):1–40. 10.1145/2543581.2543590

[10] Pastel S Chen C-H Martin L Naujoks M Petri K Witte K. Comparison of gaze accuracy and precision in real-world and virtual reality. Virtual Real. (2021) 25(1):175–89. 10.1007/s10055-020-00449-3

[11] Hussain R Chessa M Solari F. Improving depth perception in immersive Media devices by addressing vergence-accommodation conflict. IEEE Trans Visual Comput Graphics. (2024) 30(9):6334–46. 10.1109/TVCG.2023.3331902 37956016

[12] Wang Y-J Lin Y-H. Liquid crystal technology for vergence-accommodation conflicts in augmented reality and virtual reality systems: a review. Liq Cryst Rev. (2021) 9(1):35–64. 10.1080/21680396.2021.1948927

[13] Zhang Z Sternad D. Back to reality: differences in learning strategy in a simplified virtual and a real throwing task. J Neurophysiol. (2021) 125(1):43–62. 10.1152/jn.00197.2020 33146063 PMC8087380

[14] Drew L. Training for the impossible. Springer Nature. (2021) 592:4–6. 10.1038/d41586-021-00816-3

[15] Witte K Droste M Ritter Y Emmermacher P Masik S Bürger D Sports training in virtual reality to improve response behavior in karate kumite with transfer to real world. Front Virtual Real. (2022) 3:903021. 10.3389/frvir.2022.903021

[16] Zhang L Brunnett G Petri K Danneberg M Masik S Bandow N Karakter: an autonomously interacting karate kumite character for VR-based training and research. Comput Graph. (2018) 72:59–69. 10.1016/j.cag.2018.01.008

[17] Rothacher Y Nguyen A Lenggenhager B Kunz A Brugger P. Visual capture of gait during redirected walking. Sci Rep. (2018) 8(1):1–13. 10.1038/s41598-018-36035-6 30568182 PMC6299278

[18] Lee HT Kim YS. The effect of sports VR training for improving human body composition. J Image Video Proc. (2018) 2018(1):148. 10.1186/s13640-018-0387-2

[19] Bürger D Ritter Y Pastel S Sprich M Lück T Hacke M The impact of virtual reality training on learning gymnastic elements on a balance beam with simulated height. Int J Comput Sci Sport. (2022) 21(1):93–110. 10.2478/ijcss-2022-0005

[20] Pastel S. Visual perception in virtual reality and the application in sports (PhD thesis, Otto-von-Guericke University Magdeburg). Universitäts- und Landesbibliothek Sachsen-Anhalt, Halle, Germany (2021). 10.25673/58200

[21] Pastel S Chen C-H Petri K Witte K. Effects of body visualization on performance in head-mounted display virtual reality. PLoS One. (2020) 15(9):e0239226. 10.1371/journal.pone.0239226 32956420 PMC7505416

[22] Pastel S Petri K Chen CH Wiegand Cáceres AM Stirnatis M Nübel C Training in virtual reality enables learning of a complex sports movement. Virtual Real. (2023) 27(2):523–40. 10.1007/s10055-022-00679-7

[23] Appelbaum LG Erickson G. Sports vision training: a review of the state-of-the-art in digital training techniques. Int Rev Sport Exerc Psychol. (2018) 11(1):160–89. 10.1080/1750984X.2016.1266376

[24] Gerschütz B Fechter M Schleich B Wartzack S. A review of requirements and approaches for realistic visual perception in virtual reality. Proc Int Conf Eng Des. (2019) 1(1):1893–902. 10.1017/dsi.2019.195

[25] Harris DJ Buckingham G Wilson MR Vine SJ. Virtually the same? How impaired sensory information in virtual reality may disrupt vision for action. Exp Brain Res. (2019) 237(11):2761–6. 10.1007/s00221-019-05642-8 31485708 PMC6794235

[26] Hoang T Aggarwal D Wood-Bradley G Lee T-K Wang R Ferdous H A systematic review of immersive technologies for physical training in fitness and sports. 2023 IEEE International Symposium on Mixed and Augmented Reality (ISMAR). IEEE (2023). p. 611–21

[27] Mahalil I Yusof AM Ibrahim N. A literature review on the usage of technology acceptance model for analysing a virtual reality’s cycling sport applications with enhanced realism fidelity. 2020 8th International Conference on Information Technology and Multimedia (ICIMU). IEEE (2020). p. 237–42

[28] Malachi EG Tunggara R Cahyadi Y Meiliana Fajar M. A systematic literature review of virtual reality implementation in sports. 2023 International Conference on Artificial Intelligence in Information and Communication (ICAIIC). IEEE (2023). p. 382–5

[29] Putranto JS Heriyanto J Kenny AS Kurniawan A. Implementation of virtual reality technology for sports education and training: systematic literature review. Procedia Comput Sci. (2023) 216:293–300. 10.1016/j.procs.2022.12.139

[30] Richlan F Weiß M Kastner P Braid J. Virtual training, real effects: a narrative review on sports performance enhancement through interventions in virtual reality. Front Psychol. (2023) 14:1240790. 10.3389/fpsyg.2023.1240790 37928573 PMC10622803

[31] Yuqing Z Mingliang C Haoyang Z Yong Z. VR Technology and application in martial arts. 2021 IEEE 7th International Conference on Virtual Reality (ICVR). IEEE (2021). p. 240–5

[32] Mohler BJ Creem SH Thompson WB Bülthoff HH. The effect of viewing a self-avatar on distance judgments in an HMD-based virtual environment. Presence. (2010) 19(3):230–42. 10.1162/pres.19.3.230

[33] Bonfert M Lemke S Porzel R Malaka R. Kicking in virtual reality: the influence of foot visibility on the shooting experience and accuracy. 2022 IEEE Conference on Virtual Reality and 3D User Interfaces (VR). IEEE (2022). p. 711–8

[34] Caramenti M Lafortuna CL Mugellini E Abou Khaled O Bresciani J-P Dubois A. Regular physical activity modulates perceived visual speed when running in treadmill-mediated virtual environments. PLoS One. (2019) 14(6):e0219017. 10.1371/journal.pone.0219017 31242254 PMC6594642

[35] Colombo V Bocca G Mondellini M Sacco M Aliverti A. Evaluating the effects of virtual reality on perceived effort during cycling: preliminary results on healthy young adults. 2022 IEEE International Symposium on Medical Measurements and Applications (MeMeA). IEEE (2022). p. 1–6

[36] Covaci A Olivier A-H Multon F. Visual perspective and feedback guidance for VR free-throw training. IEEE Comput Grap Appl. (2015) 35(5):55–65. 10.1109/MCG.2015.95 26416362

[37] Dhawan A Cummins A Spratford W Dessing JC Craig C. Development of a novel immersive interactive virtual reality cricket simulator for cricket batting. In: Chung P Soltoggio A Dawson CW Meng Q Pain M, editors. Proceedings of the 10th International Symposium on Computer Science in Sports (ISCSS). Cham: Springer International Publishing (2016). p. 203–10. (Advances in Intelligent Systems and Computing).

[38] Drew SA Awad MF Armendariz JA Gabay B Lachica IJ Hinkel-Lipsker JW. The trade-off of virtual reality training for dart throwing: a facilitation of perceptual-motor learning with a detriment to performance. Front Sports Act Living. (2020) 2:59. 10.3389/fspor.2020.00059 33345050 PMC7739782

[39] Finnegan SL Dearlove DJ Morris P Freeman D Sergeant M Taylor S Breathlessness in a virtual world: an experimental paradigm testing how discrepancy between VR visual gradients and pedal resistance during stationary cycling affects breathlessness perception. PLoS One. (2023) 18(4):e0270721. 10.1371/journal.pone.0270721 37083693 PMC10120935

[40] Geisen M Nicklas A Baumgartner T Klatt S. Extended reality as a training approach for visual real-time feedback in golf. IEEE Trans Learning Technol. (2024) 17:642–52. 10.1109/TLT.2023.3322660

[41] Godse A Khadka R Banic A. Evaluation of visual perception manipulation in virtual reality training environments to improve golf performance. 2019 IEEE Conference on Virtual Reality and 3D User Interfaces (VR). IEEE (2019). p. 1807–12

[42] Gong U Jia H Wang Y Tang T Xie X Wu Y. Volleynaut: pioneering immersive training for inclusive sitting volleyball skill development. 2024 IEEE Conference Virtual Reality and 3D User Interfaces (VR). IEEE (2024). p. 1022–32

[43] Harris DJ Buckingham G Wilson MR Brookes J Mushtaq F Mon-Williams M The effect of a virtual reality environment on gaze behaviour and motor skill learning. Psychol Sport Exerc. (2020) 50:101721. 10.1016/j.psychsport.2020.101721

[44] Harris DJ Wilson MR Vine SJ. The functional role of visual information and fixation stillness in the quiet eye. PLoS One. (2023) 18(11):e0293955. 10.1371/journal.pone.0293955 37930988 PMC10627465

[45] Hosp B Schultz F Kasneci E Höner O. Expertise classification of soccer goalkeepers in highly dynamic decision tasks: a deep learning approach for temporal and spatial feature recognition of fixation image patch sequences. Front Sports Act Living. (2021) 3:692526. 10.3389/fspor.2021.692526 34381997 PMC8350442

[46] Hülsmann F Frank C Senna I Ernst MO Schack T Botsch M. Superimposed skilled performance in a virtual mirror improves motor performance and cognitive representation of a full body motor action. Front Robot AI. (2019) 6:43. 10.3389/frobt.2019.00043 33501059 PMC7805859

[47] Ishibe K Aihara S Hayashi Y Iwata H. The development of an immersive three-dimensional virtual reality system for identifying hand–eye coordination in badminton. 2020 IEEE International Conference on Systems, Man, and Cybernetics (SMC). IEEE (2020). p. 1778–84

[48] Klatt S Ford PR Smeeton NJ. Attentional and perceptual asymmetries in an immersive decision-making task. Atten Percept Psychophys. (2020) 82(4):1847–57. 10.3758/s13414-019-01935-w 31808113

[49] Ku C Weng J-J Wang Y-H Wu D-X Lau Y-M Tsai W-L Table tennis skill learning in VR with step by step guides using forehand drive as a case study. 2022 IEEE International Conference on Artificial Intelligence and Virtual Reality (AIVR). IEEE (2022). p. 275–82

[50] Li H Mavros P Krukar J Hölscher C. The effect of navigation method and visual display on distance perception in a large-scale virtual building. Cogn Process. (2021) 22(2):239–59. 10.1007/s10339-020-01011-4 33564939 PMC8179918

[51] Limballe A Kulpa R Vu A Mavromatis M Bennett SJ. Virtual reality boxing: gaze-contingent manipulation of stimulus properties using blur. Front Psychol. (2022) 13:902043. 10.3389/fpsyg.2022.902043 36248589 PMC9557204

[52] Loiseau Taupin M Romeas T Juste L Labbé DR. Exploring the effects of 3D-360°VR and 2D viewing modes on gaze behavior, head excursion, and workload during a boxing specific anticipation task. Front Psychol. (2023) 14:1235984. 10.3389/fpsyg.2023.1235984 37680243 PMC10481868

[53] Luke IM Neumann DL Stainer MJ Potter LE Moffitt RL. Eye-gaze behaviour of expert and novice surfers in a simulated surf environment. Psychol Sport Exerc. (2022) 62:102221. 10.1016/j.psychsport.2022.102221

[54] Lynch SD Olivier A-H Bideau B Kulpa R. Detection of deceptive motions in rugby from visual motion cues. PLoS One. (2019) 14(9):e0220878. 10.1371/journal.pone.0220878 31518358 PMC6743770

[55] Morice AHP Hadatine I Marot J. A framework for optimizing AI-based virtual reality: a use case in sport sciences. 2023 IEEE 25th International Workshop on Multimedia Signal Processing (MMSP). IEEE (2023). p. 1–6

[56] Nasu D Baba T Imamura T Yamaguchi M Kitanishi Y Kashino M. Virtual reality perceptual training can improve the temporal discrimination ability of swinging during softball batting. Front Sports Act Living. (2024) 6:1332149. 10.3389/fspor.2024.1332149 38450282 PMC10915064

[57] Oagaz H Schoun B Choi M-H. Performance improvement and skill transfer in table tennis through training in virtual reality. IEEE Trans Vis Comput Graph. (2022) 28(12):4332–43. 10.1109/TVCG.2021.3086403 34081582

[58] Okada Y Seo C Miyakawa S Taniguchi M Kanosue K Ogata H Virtual ski training system that allows beginners to acquire ski skills based on physical and visual feedbacks. 2023 IEEE/RSJ International Conference on Intelligent Robots and Systems (IROS). IEEE (2023). p. 1268–75

[59] Pastel S Petri K Bürger D Marschal H Chen C-H Witte K. Influence of body visualization in VR during the execution of motoric tasks in different age groups. PLoS One. (2022) 17(1):e0263112. 10.1371/journal.pone.0263112 35077512 PMC8789136

[60] Perrin T Kerherve HA Faure C Sorel A Bideau B Kulpa R. Enactive approach to assess perceived speed error during walking and running in virtual reality. 2019 IEEE Conference on Virtual Reality and 3D User Interfaces (VR). IEEE (2019). p. 622–9

[61] Petri K, Witte K, Bandow N, Emmermacher P, Masik S, Dannenberg M

[62] Rojas Ferrer CD Shishido H Kitahara I Kameda Y. Read-the-game: system for skill-based visual exploratory activity assessment with a full body virtual reality soccer simulation. PLoS One. (2020) 15(3):e0230042. 10.1371/journal.pone.0230042 32182621 PMC7077991

[63] Romeas T Chaumillon R Labbé D Faubert J. Combining 3D-MOT with sport decision-making for perceptual-cognitive training in virtual reality. Percept Mot Skills. (2019) 126(5):922–48. 10.1177/0031512519860286 31272277

[64] Runswick OR. Player perceptions of face validity and fidelity in 360-video and virtual reality cricket. J Sport Exerc Psychol. (2023) 45(6):347–54. 10.1123/jsep.2023-0122 37935172

[65] Sigrist R Rauter G Marchal-Crespo L Riener R Wolf P. Sonification and haptic feedback in addition to visual feedback enhances complex motor task learning. Exp Brain Res. (2015) 233(3):909–25. 10.1007/s00221-014-4167-7 25511166

[66] Soltani P Morice AHP. A multi-scale analysis of basketball throw in virtual reality for tracking perceptual-motor expertise. Scand J Med Sci Sports. (2023) 33(2):178–88. 10.1111/sms.14250 36315055 PMC10100508

[67] Tian F Ni S Zhang X Chen F Zhu Q Xu C Enhancing tai chi training system: towards group-based and hyper-realistic training experiences. IEEE Trans Vis Comput Graph. (2024) 30(5):2713–23. 10.1109/TVCG.2024.3372099 38457324

[68] Tian F Zou J Li K Li Y. Kung fu metaverse: a movement guidance training system. IEEE Trans Learning Technol. (2023) 16(6):1082–95. 10.1109/TLT.2023.3317945

[69] Tsai W-L Pan T-Y Hu M-C. Improve immersion in virtual reality-based basketball training by haptic feedback. Proceedings of the 2022 ACM International Joint Conference on Pervasive and Ubiquitous Computing. New York, NY, USA: ACM (2022). p. 524–8

[70] Valkanidis TC Craig CM Cummins A Dessing JC. A goalkeeper’s performance in stopping free kicks reduces when the defensive wall blocks their initial view of the ball. PLoS One. (2020) 15(12):e0243287. 10.1371/journal.pone.0243287 33382753 PMC7774851

[71] Vargas González AN Kapalo K Koh S LaViola J. Exploring the virtuality Continuum for Complex rule-set education in the context of soccer rule comprehension. MTI. (2017) 1(4):30. 10.3390/mti1040030

[72] Waltemate T Senna I Hülsmann F Rohde M Kopp S Ernst M The impact of latency on perceptual judgments and motor performance in closed-loop interaction in virtual reality. Proceedings of the 22nd ACM Conference on Virtual Reality Software and Technology. New York, NY, USA: ACM (2016). p. 27–35

[73] Weng J-J Wang Y-H Ku C Wu D-X Lau Y-M Tsai W-L Assist home training table tennis skill acquisition via immersive learning and web technologies. 2022 IEEE Conference on Virtual Reality and 3D User Interfaces Abstracts and Workshops (VRW). IEEE (2022). p. 804–5

[74] Pastel S Bürger D Chen CH Petri K Witte K. Comparison of spatial orientation skill between real and virtual environment. Virtual Real. (2022) 26(1):91–104. 10.1007/s10055-021-00539-w

[75] Li T Wang H Fan D Wang D Yin L Lan Q. Research on virtual skiing system based on harmonious human-computer interaction. 2022 International Conference on Virtual Reality, Human-Computer Interaction and Artificial Intelligence (VRHCIAI). IEEE (2022). p. 106–10

